# Lower multisensory temporal acuity in individuals with high schizotypal traits: a web-based study

**DOI:** 10.1038/s41598-022-06503-1

**Published:** 2022-02-17

**Authors:** Gianluca Marsicano, Filippo Cerpelloni, David Melcher, Luca Ronconi

**Affiliations:** 1 School of Psychology, Vita-Salute San Raffaele University, Milan, Italy; 2 Division of Neuroscience, IRCCS San Raffaele Scientific Institute, Milan, Italy; 3 Center for Mind/Brain Sciences and Department of Psychology and Cognitive Science, University of Trento, Rovereto, Italy; 4 Laboratory of Biological Psychology, Department of Brain and Cognition, Leuven Brain Institute, KU Leuve, Leuven, Belgium; 5 Institute of Research in Psychology (IPSY) & Institute of Neuroscience (IoNS)-University of Louvain (UCLouvain), Leuven, Belgium; 6 Psychology Program, Division of Science, New York University Abu Dhabi, Abu Dhabi, United Arab Emirates

**Keywords:** Human behaviour, Perception

## Abstract

Natural events are often multisensory, requiring the brain to combine information from the same spatial location and timing, across different senses. The importance of temporal coincidence has led to the introduction of the temporal binding window (TBW) construct, defined as the time range within which multisensory inputs are highly likely to be perceptually bound into a single entity. Anomalies in TBWs have been linked to confused perceptual experiences and inaccurate filtering of sensory inputs coming from different environmental sources. Indeed, larger TBWs have been associated with disorders such as schizophrenia and autism and are also correlated to a higher level of subclinical traits of these conditions in the general population. Here, we tested the feasibility of using a web-based version of a classic audio-visual simultaneity judgment (SJ) task with simple flash-beep stimuli in order to measure multisensory temporal acuity and its relationship with schizotypal traits as measured in the general population. Results show that: (i) the response distribution obtained in the web-based SJ task was strongly similar to those reported by studies carried out in controlled laboratory settings, and (ii) lower multisensory temporal acuity was associated with higher schizotypal traits in the “cognitive-perceptual” domains. Our findings reveal the possibility of adequately using a web-based audio-visual SJ task outside a controlled laboratory setting, available to a more diverse and representative pool of participants. These results provide additional evidence for a close relationship between lower multisensory acuity and the expression of schizotypal traits in the general population.

## Introduction

In daily life our brains constantly encode, filter and integrate information from different sensory modalities, in such a way as to give order to our perceptual experience. This process, called multisensory integration, is an essential feature of human cognition which allows the input in the environment from different sensory modalities to be correctly integrated into a coherent, unitary percept, while also correctly segmenting sensory information from different events as separate and unique events^1–3^. Multisensory perception, then, forms an important foundation in constructing our perceptual experience in terms of specific objects, events and social interactions.

A key aspect of multisensory processing is to correctly perceive information coming from different sensory modalities about the same object/event as belonging to a single event, rather than as separate events^4^, facilitating the skills of detection^5,6^ and localization^7^, in order to adapt behavior to environmental demands^8^. Multisensory integration is not limited only to the processing of simple sensory stimuli, but yields beneficial cascade effects also in higher-order cognitive and social processes, which translate into an improvement of the functions such as speech perception^9,10^ and complex verbal and non-verbal problem solving, reasoning and thoughts^11^.

This integrative ability is strongly dependent on the spatiotemporal relationship and on the physical characteristics of stimuli of different sensory origin^12,13^, factors that act as statistical indices of the possibility that the stimuli are paired together. Such regularities increase the likelihood that stimulation to different senses comes from the same spatiotemporal event.

A greater spatial and temporal proximity of the stimuli facilitates multisensory interactions^14^. The importance of these factors is evident at various levels of investigation, as shown by numerous neuroimaging^15–19^ and psychophysical^20–29^ studies. The spatial position of stimuli influences the perception of simultaneity, such that auditory and visual input are more likely to be integrated into a single percept and judged as simultaneous, when presented in congruent spatial positions, rather than in different locations^20^. Conversely, subjects restrict their temporal acuity, becoming better in discriminating and detecting asynchrony, when the stimuli appear in different spatial positions^20,27^. Such spatial effects that influence multisensory perception processes are evident at the neurophysiological, neuroimaging and behavioral level^30–33^. Moreover, the temporal distance between stimuli of different sensory modalities determines the occurrence of the multisensory integration process, reflecting the key role of temporal processing for accurate multisensory integration^20,27^. Critically, multisensory integration not only occurs when the two stimuli are presented perfectly synchronously, but can also occur when stimuli are slightly asynchronous^34,35^. Given that auditory and visual stimuli are processed with different neural mechanisms and timing^36–38^, such temporal “tolerance” allows them to be integrated into a single percept if they originate from the same source^39,40^. At the same time, such temporal tolerance should be limited to provide order to the perceptual experience, for example to result in an accurate differentiation of auditory and visual input coming from different events^41^. Perceptual binding of audiovisual stimuli is more likely to occur if they are presented in proximity within a limited temporal interval, a process that is operationalized in the construct of the “temporal binding window” (TBW)^8,27,42,43^. The width of this temporal window is commonly used as a proxy measure for multisensory temporal acuity. The TBW is a probabilistic construct, defined by the range of stimulus onset asynchronies (SOAs) in which stimuli of different sensory modalities are likely to be perceptually paired.

Interestingly, perceiving two stimuli as simultaneous does not require that they are presented at the same time and perceived simultaneity can even be greater when a stimulus from one modality precedes the stimulus from the other modality. Experimental manipulation of the “leading” sensory modality, that is the order in which the audiovisual stimuli are presented, influences the judgment of simultaneity^27,43,44^. At the behavioral level, these asymmetries in the distribution obtained from the simultaneity rate indicate a narrower size of the binding window for Auditory‐Leading (AL) as compared to Visual‐Leading (VL) trials^44^. At the neural level, these asymmetries are characterized by two distinct patterns of brain activity, which reveal that potentially different mechanisms support different multisensory processes^45^.

Using similar paradigms recent studies have revealed that, within the general population, TBW in multisensory processing is not homogeneous, but is characterized by a high inter-individual variability^46^. The current idea is that a certain degree of tolerance of time delays between the presentation of stimuli from different sensory modalities is adaptive^47–49^. Such adaptive temporal tolerance would make us insensitive to small differences in the arrival time of signals to different sensory modalities and has beneficial effect in complex cognitive functions, like speech perception, which require continuous binding between visual and auditory information^39^. However, when this tolerance becomes excessively liberal, it may result in an ambiguous perceptual experience or sensory overload^46,48,49^, in a confused sense of self^50^ or even in hallucinations^51^. This reduced precision in the multisensory integration process is typical in the clinical population affected by Schizophrenia (SCZ), where an enlarged TBW is associated with socio-cognitive and perceptual differences^52^, resulting in an aberrant synchrony perception of audiovisual stimuli that likely determines an ambiguous and imprecise perceptual experience^53–56^. Moreover, sub-clinical traits of SCZ are also present in the non-clinical population^57^, with higher traits reflecting a higher prevalence and intensity of behavioral characteristics associated with SCZ. Indeed, individuals with higher traits of SCZ show perceptual and cognitive deficits, such as in early sensorimotor processing^58,59^, working memory^60^, executive functions^61^ and attention^62^.

Interestingly, deficits in the temporal processes of multisensory integration have recently been identified also in the non-clinical population with higher schizotypal traits^50,63^. For example, one recent study has shown that a lower temporal acuity in multisensory integration is correlated to a higher level of schizotypal symptomatology, and that this relationship is specific for the cognitive-perceptual domain^64^. These studies suggest the need to extend the investigation of multisensory temporal processing anomalies also to the general population with a varying degree of schizotypal traits.

In the current study we have employed an online version of the Simultaneity Judgment (SJ) task, which is one of the main experimental paradigms developed to investigate TBW, in which participants are asked to judge the perceived simultaneity of visual and auditory stimuli presented in congruent or incongruent spatial position across different SOAs^20,65,66^. The rate of perceived simultaneity responses across different SOAs is used to estimate the width of the TBW. Hence, the individual's TBW indicates the time range for which subjects show a high probability of judging two stimuli as simultaneous, and is typically defined as a function of the time interval between the 75% thresholds observed in AL and VL trials^52^.

The first aim of this study was to demonstrate the possibility of conducting a typical lab-based psychophysical task through a web-based platform, in a less controlled setting. The need and the possibility of adapting lab-based experiments to online context derives from the advantages offered by this system. Indeed, it is possible to make research more accessible to a greater number of participants, to increase their diversity within the recruited sample (such as: age, gender, origin, culture and social status) and to optimize the efficiency and timing of test administration^67–69^. Importantly, the use of this system allows recruiting larger samples that does not require a higher workload^70–72^. In line with previous lab-based evidence in the literature^20,65^ we expect that the multisensory integration process of audiovisual stimuli would vary according to the SOAs. Increasing the temporal synchrony of audiovisual stimuli would lead to a high degree of perceived simultaneity of audiovisual stimuli compared to trials in which the SOA is larger. We also expected a generally greater multisensory temporal acuity when stimuli were presented in the same spatial position, rather than in different locations. Based on recent evidence^44,45^, we also focused our attention on the possible asymmetries in the multisensory integration processes deriving from the leading sense (AL vs VL).

Alongside the web-based behavioral SJ task, participants were asked to complete the Schizotypal Personality Questionnaire (SPQ)^73,74^, to analyze the relationship between schizotypal traits in the general population and multisensory temporal acuity. Based on recent evidence^50,52,56,64^, we hypothesized that a lower multisensory temporal acuity—i.e. an enlarged TBW—would correlate with higher levels of schizotypal traits.

It is worth noting that some theories of schizophrenia suggest that development of clinical levels of SCZ is linked to both genetics and to environmental stressors, with less stressors potentially leading to schizotypy rather than full schizophrenia^75,76^. If so, then widespread screening of schizotypal traits and perceptual/cognitive functions using simple web-based tasks might be useful for identifying markers for at-risk individuals.

## Methods

### Participants

A total of 55 participants took part initially in the study. All were volunteers and presented normal or corrected to normal vision and hearing. Four subjects were excluded from analyses due to inability to do the task, as they did not reach a 50% mean rate of simultaneity in at least one of the conditions. The final sample of participants included in the analyses comprised 51 participants (26 F, mean age = 23.9, SD = 3.26). All participants were provided with a document containing details about the procedure for completing the questionnaire and the web-based version of the audiovisual SJ task. In particular, we underlined the importance of following the instructions we provided for an optimal execution of the online task, where we stressed the importance of sitting in a quiet room, using headphones/earbuds at a comfortable volume. Participants did not receive specific instruction concerning screen brightness, although this aspect could be considered secondary as stimuli were created at high contrast. We specified that the link could be used only from a PC and not from mobile devices. Experiments were conducted in accordance with the Declaration of Helsinki and the study was approved by the Ethical Committee of the University of Trento. All participants gave their informed consent.

### Questionnaire for schizotypal traits

Alongside the behavioral experiment, participants were asked to complete the Schizotypal Personality Questionnaire (SPQ)^73,74^ presented online through the Google Forms platform. This self-report questionnaire aimed at estimating the presence of schizotypal traits and is composed of 74 questions in which participants are asked to answer questions regarding different aspects of their personality, in addition to questions concerning sensorial experiences and beliefs, with “Yes'' or “No” statements. In particular, the test contains nine subscales, divided into three components: Cognitive-Perceptual (subscales: ideas of reference, unusual perceptual experiences, magical thinking, suspiciousness), Disorganization (subscales: odd speech, odd or eccentric behavior) and Interpersonal (subscales: excessive social anxiety, no close friends, constricted affect, suspiciousness).

### Online audiovisual simultaneity judgement (SJ) task

The task was crated with PsychoPy^77,78^ and administered using *Pavlovia* (https://pavlovia.org/), a web-based platform for the presentation of psychophysics experiments via common web browsers. This allowed us to collect remotely data for the SJ task. The audiovisual stimulus pairs were initially created in the form of videos at high contrast using Psychtoolbox on Matlab 2020a and then manipulated with Blender (https://www.blender.org/) in order to create the different SOA conditions. This strategy was chosen after pilot testing because it was the most reliable across operating systems (OS). We were able to collect directly from PsychoPy/Pavlovia information about the type of OS used by the participants (MacOs = 33 participants, Windows = 21 participants, Linux = 1 participant). We asked subjects to run the experiment using Firefox as a web browser; if they tried to use other web browsers an error message appeared at the beginning of the experiment and the task was aborted. Firefox was chosen after extensive pilot observation because it was the most reliable in terms of stimuli presentation across the different OS. All participants performed the task with 60 Hz a refresh rate.

**Figure 1:**
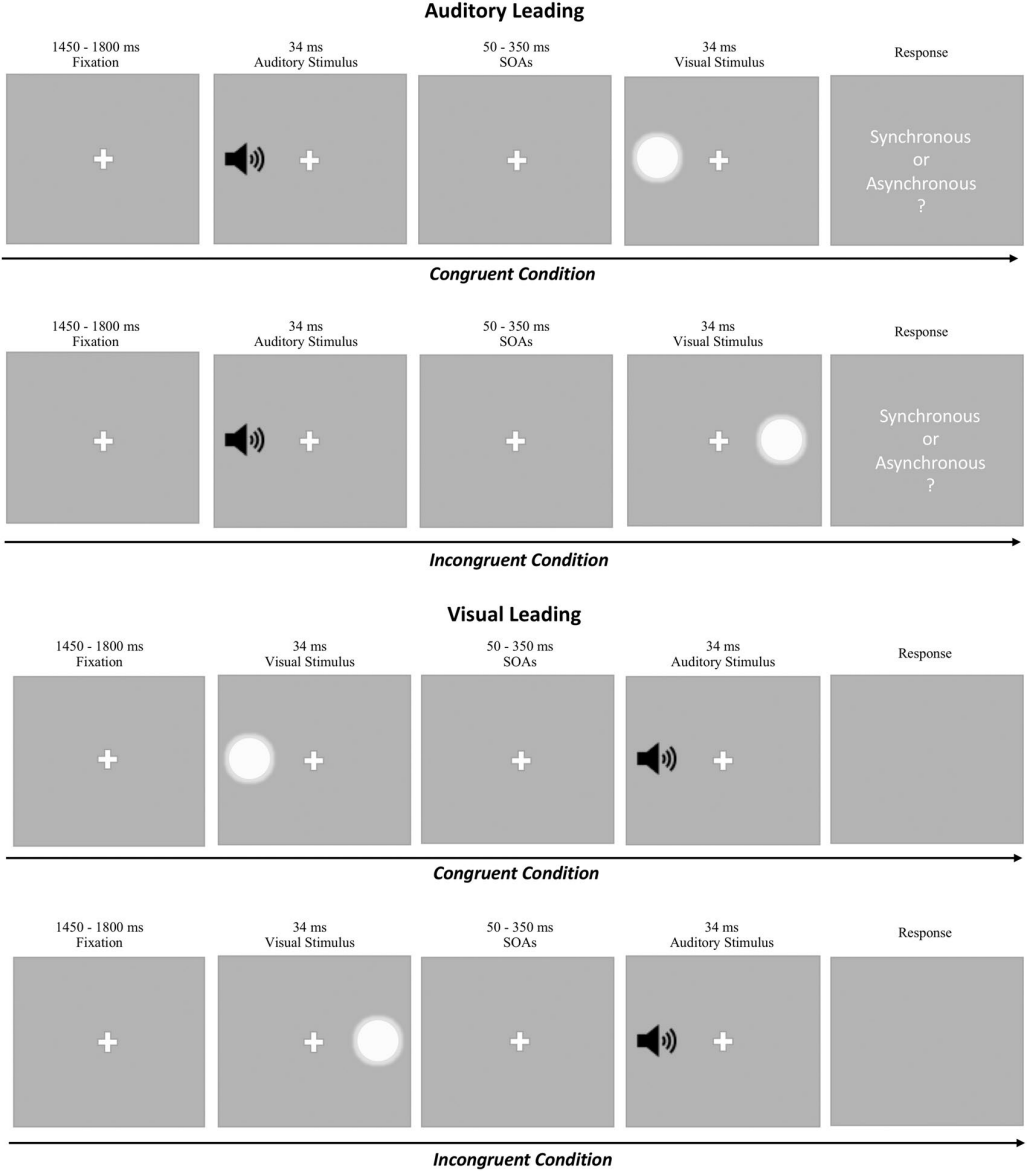
Schematic representation of the web-based Simultaneity Judgment task. In each trial a fixation point is presented at the center of the screen for a variable time between 1450 and 1800 ms before the appearance of the first stimulus. A fixed array of SOAs between the first and second stimulus was used across trials (± 350, ± 300, ± 250, ± 200, ± 150, ± 100, ± 50, 0 ms). Trials were counterbalanced between the following two conditions: (**A**) Auditory Leading (AL) trials: when the auditory stimulus was followed by the visual stimulus, (**B**) Visual Leading (VL trials): when the visual stimulus was followed by the auditory stimulus. The stimuli were presented alternatively on the left or right side of the screen and auditory stimuli on either the left or right headphones/earphones channel, leading to a spatially congruent or incongruent stimulus presentation.

To investigate spatial position effects, visual stimuli were presented either on the left or right side of the screen and auditory stimuli on either the left or right headphones/earbuds channel. The location of visual and auditory stimuli for any given trial was not predictable for the participant. To explore the extent of the TBW, a fixed array of SOAs was used (± 350, ± 300, ± 250, ± 200, ± 150, ± 100, ± 50, 0 ms) presenting either the auditory (AL trials) or the visual (VL trials) stimulus first (Fig. 1). The two order conditions were balanced across trials. Assuming participants kept the recommended distance of 50 cm from the screen, the visual stimulus employed was a white Gaussian blob with a diameter of 5° of visual angle, presented at approximately 18° of eccentricity from the central fixation point on a middle grey background. All visual stimuli lasted for a total of ~ 33 ms, equivalent to 2 frames at 60 Hz (the refresh rate of common PCs/laptops). As part of the data collection process, the refresh rate of the monitor/display was recorded for each participant. After data collection, we confirmed that all participants performed the task at this refresh rate with no exceptions. The auditory stimulus was a pure tone at 750 Hz with a duration of ~ 33 ms, to match the visual counterpart.

The total amount of trials administered for each participant were 252, consisting of 12 practice trials and 240 real trials. The 240 experimental trials were presented in a single block, which included 8 repetitions for each combination of SOAs and spatial congruency. The order of trials was chosen randomly across participants.

In each trial a fixation point was presented at the center of the screen for a fixed time of 1300 ms and a variable time between 150 and 500 ms before the appearance of the first stimulus. After each trial, participants were asked to rate the simultaneity of auditory and visual stimuli on a 5-point scale providing their responses from a keyboard, with rating 1–2 indicating the presence of simultaneity (1 = certainly synchronous; 2 = probably synchronous), ratings 4–5 indicating the absence of simultaneity (4 = probably asynchronous; 5 = certainly asynchronous), and rating 3 indicating a ‘not sure/not seen’ response. The latter was later discarded from analyses as non-informative. The response was given with no time constraints and the total time taken by the participants to complete the experiment was ~ 20 min.

After data collection, we realized that—possibly because of the limited number of trials—the individual distribution of ‘probably’/‘certainly’ responses did not allow us to perform a threshold estimation as a function of the level of confidence, and for this reason we opted for performing an analysis based on a 3-point scale, combining the different levels of confidence into one perceptual outcome (i.e. synchronous/asynchronous), and excluding trials with unclear subjective outcomes.

### Data analysis

Prior to the data analysis, variables were plotted and checked for the normality of the distribution using Shapiro–Wilk tests. For the simultaneity rate in AL and VL trials and AL threshold, Shapiro–Wilk tests were not significant (AL simultaneity rate: p = 0.813; VL simultaneity rate: p = 0.570; AL threshold: p = 0.527), indicating normal distributions. By contrast, the VL threshold was non-normally distributed (Shapiro–Wilk: p = 0.001).

We first performed a repeated measures Analysis of Variance (ANOVA) on the rate of synchronous responses with the aim of testing whether performance was influenced by the spatial congruency factor (two levels: congruent vs. incongruent) and SOAs (fifteen levels: − 350, − 300, − 250, − 200, − 150, − 100, − 50, 0, + 50, + 100, + 150, + 200, + 250, + 300, + 350), which were used as within-subjects factors. The Greenhouse–Geisser correction was applied in cases in which the sphericity assumption was violated.

In a second step, we obtained the individual 75% thresholds values from the fitting of the psychometric logistic curve for each subject, separately for the AL and the VL condition, and for each spatial congruency condition (congruent vs. incongruent). The psychometric curve was a logistic function fit to the participant responses for the different SOAs. In the case of AL, we determined the absolute value of the threshold (that is normally negative, because is extracted from the psychometric curve of the left side), while the value of the VL threshold is typically a positive value, extracted from the psychometric curve of the right side. This estimation of the AL and VL threshold was calculated for every participant individually. Specifically, for each participant we employed a logistic equation and a non-linear least squares method to fit the proportion of simultaneity rate reported to the SJ task as a function of SOA. The formula used was the following: *y* = *1/(1* + *exp (b* × *(t – x ))).* In this equation, x represents the SOA between audio and visual stimuli and y represents the proportion of simultaneity responses. The lower y bound was set at 0 and the higher y bound was set at 1 (y = 0 means that audiovisual stimuli were never perceived as synchronous, and y = 1 means that they were always perceived as synchronous). The only free parameters of the function were b (the function slope) and t (the 75% threshold), which were restricted to assume positive values above zero. Both AL and VL curves were fitted using also the data point corresponding to SOA = 0 ms. This choice has been made following the previous literature^42^.

We performed two additional repeated measures ANOVAs using as dependent variables both the overall simultaneity rate (i.e. averaged across negative/AL and positive/VL SOAs, see “Data analysis” section) and 75% thresholds with the aim of testing whether performance was influenced by the spatial congruency factor (two levels: congruent vs. incongruent) and leading sense factor (two levels: AL vs. VL), which were used as within-subjects factors. The Greenhouse–Geisser correction was applied in cases where the sphericity assumption was violated.

With the purpose of testing interindividual differences in multisensory temporal acuity and as a function of schizotypal traits, following previous studies^14,52^ we additionally calculated the width of the TBW as the sum for each participant of the absolute (non-negative) 75% threshold values for AL and VL trials.

Measures derived from the SJ task (i.e. simultaneity rate, thresholds/TBW obtained from the psychometric fitting) and the scores obtained in the SPQ (i.e. the three subscales) were analyzed with the Pearson Correlation Coefficient in order to test the relation between schizotypal traits and multisensory temporal acuity. We performed the Benjamini–Hochberg false discovery rate correction for the multiple comparisons (for the 4 different SPQ scores [Total, Cognitive-Perceptual, Disorganized, Interpersonal]) on each of the two experimental measures of interest (simultaneity rates and TBW). It is worth noting that by taking the raw threshold values from VL trials and the absolute thresholds values from AL trials we could directly compare multisensory temporal acuity between the different experimental conditions (e.g. spatially congruent vs. incongruent; AL trials vs. VL trials). Moreover, by doing so we had a common measure of temporal acuity for the correlational analyses. Thus, hereafter higher threshold values will always indicate that the 75% of perceived simultaneity reports are achieved at larger SOAs between cross-modal stimuli, indicating a reduced temporal acuity (i.e. a wider TBW).

## Results

### Simultaneity judgment task

**Figure 2:**
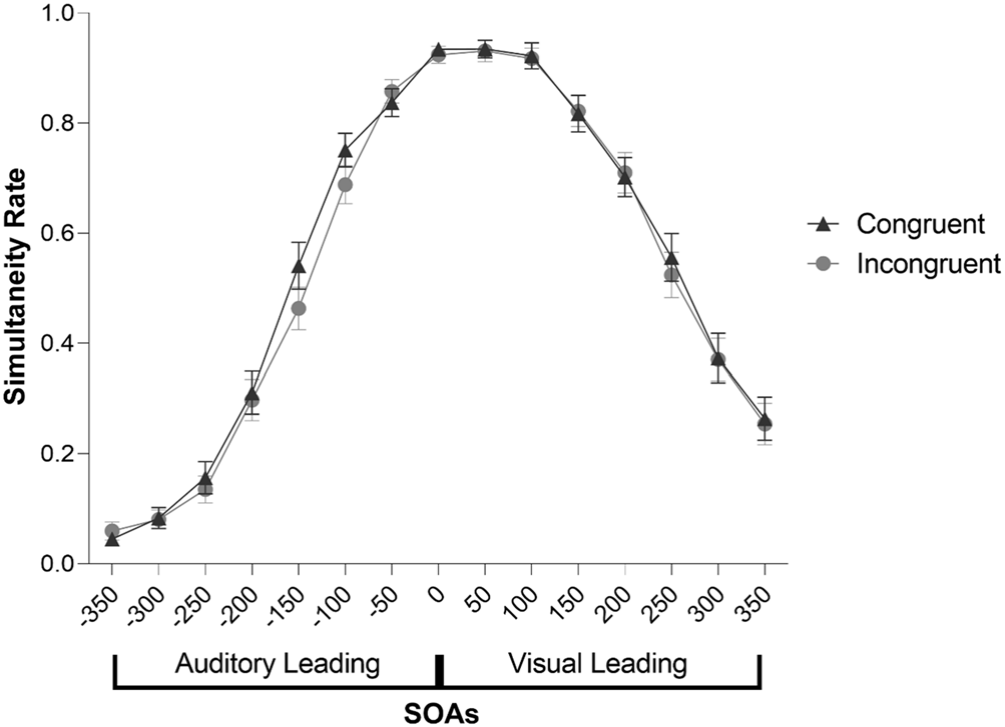
Repeated measures ANOVA performed on simultaneity rate revealed a significant main effect of SOA, revealing that the variability of the width of the time interval between the two stimuli influenced performance. This analysis did not show a significant main effect of the spatial congruency nor an interaction between spatial congruency and SOA. The error bars indicate the standard error of the mean (SEM).

As expected, the width of the time interval between the two stimuli influenced performance (Fig. 2). The first ANOVA done on simultaneity rate with the aim of testing whether performance was influenced by the spatial congruency factor and SOAs showed, indeed, a significant main effect of SOA (F(3.849, 192.448) = 184.25, p < 0.001, η^2^p = 0.787); on the contrary, the main effect of spatial congruency (F(1, 50) = 2.22, p = 0.142, η^2^p = 0.043) and the interaction between spatial congruency and SOA (F(14, 700) = 1.140, p = 0.318, η^2^p = 0.022) were not significant.

**Figure 3:**
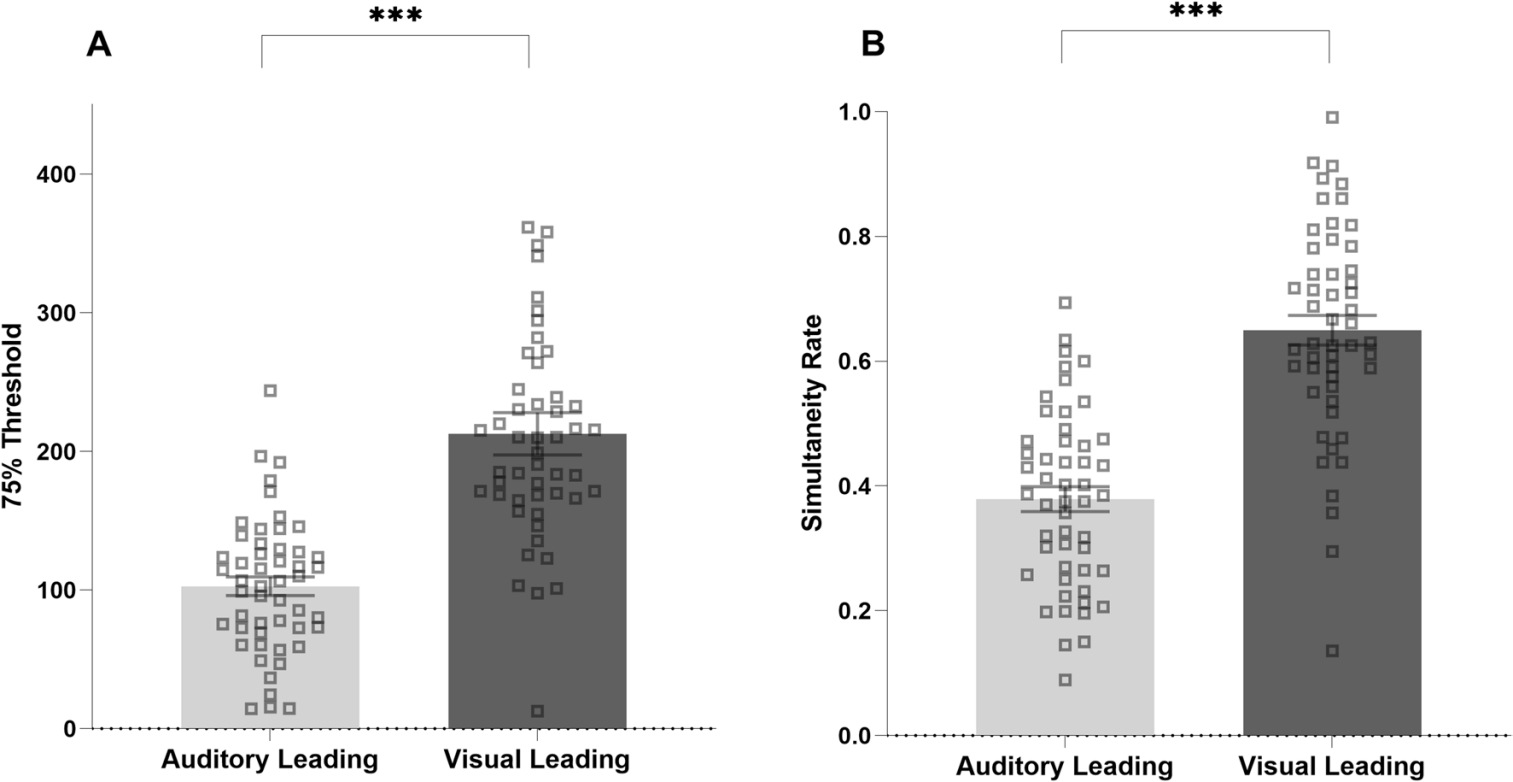
Repeated measures ANOVA performed on threshold (**A**) and simultaneity rate (**B**) revealed a significant main effect of leading sense, suggesting a higher multisensory temporal precision when audition is the leading sense. The error bars indicate the standard error of the mean (SEM). *** = p<.001.

The second ANOVA performed on simultaneity rate with the aim of testing whether performance was influenced by the spatial congruency factor and leading sense confirmed a significant main effect of leading sense (F(1, 50) = 97.538, p < 0.001, η^2^p = 0.622; see Fig. 3A). In contrast, the main effect of spatial congruency (F(1, 50) = 1.958, p = 0.168, η^2^, p = 0.001) and the interaction between spatial congruency and leading sense (F(1, 50) = 1.113, p = 0.296, η^2^p = 0.004) were not significant. A similar ANOVA performed on 75% threshold confirmed a significant main effect of leading sense (F(1, 50) = 29.537, p < 0.001, η^2^p = 0.259; see Fig. 3B), and again did not show a significant main effect of spatial congruency (F(1, 50) = 0.080, p = 0.789, η^2^p = 0.001) nor an interaction between spatial congruency and leading sense (F(1, 50) = 1.392, p = 0.244, η^2^p = 0.004). Overall, the pattern of results is in line with a generally higher multisensory temporal precision (i.e. lower thresholds/simultaneity rates) when audition is the leading sense.

The results of our study could be compared with other results obtained from an SJ task in a classic laboratory setting. The study by Fenner and colleagues^63^, in particular, is suitable to this aim since is largely comparable to the present study in terms of SOAs employed and because the auditory presentation was made also in that case with headphones/earbuds. Differently from the present study, however, Fenner and colleagues^63^ did not employ an AL condition but tested only VL trials. In our study, the observed mean simultaneity rate at each SOAs in the VL condition (averaged between spatially congruent and incongruent trials) was as follows: 50 ms = 0.93, 100 ms = 0.92, 150 ms = 0.82, 200 ms = 0.69, 250 ms = 0.54, 300 ms = 0.37, 350 ms = 0.26. Fenner and colleagues^63^ reported the following results (estimated from the figure on p. 6): 50 ms $\cong$ 0.87, 100 ms $\cong$ 0.82, 150 ms $\cong$ 0.78, 200 ms $\cong$ 0.70, 250 ms $\cong$ 0.64, 300 ms $\cong$ 0.54, 350 ms $\cong$ 0.50.

In addition, the parameters of the psychometric curves obtained in our study appear to be in line with the results obtained in a lab-based context. Indeed, Fenner and colleagues^63^ reported an average 75% threshold in VL trials of ~ 200 ms (as estimated from their plots; AL was not tested). Similarly, also Powers and colleagues^24^, who investigated the plasticity of TBW after training, reported before training 75% threshold values in VL trials of ~ 200 ms and of ~ 220 ms (as estimated from their plots) in two sample of participants who received different training protocols. The same authors reported 75% threshold values in AL trials of 100 ms and of 175 ms before different training protocols. In our web-based study, the average 75% threshold (disregarding spatial congruency) obtained was 212.7 ms in VL trials and 102.1 ms in AL trials, demonstrating a high similarity with some previous lab-based studies in the literature.

### Correlation between audio-visual temporal acuity and schizotypal traits

**Figure 4:**
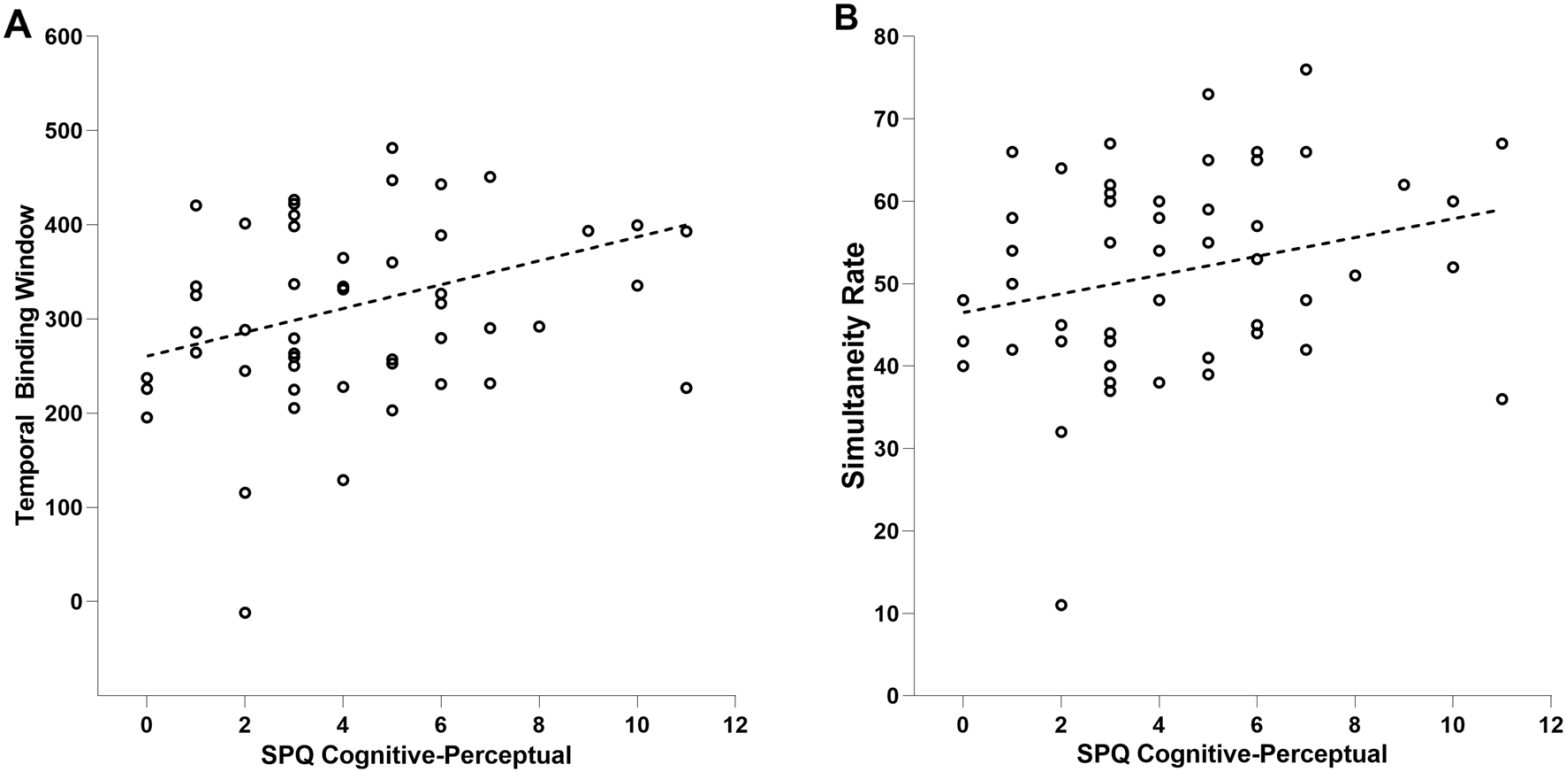
The figures highlight the associations revealed by the Pearson correlation coefficient between the measures of the web-based SJ task and schizotypal traits as measures by the SPQ. (**A**) The figure shows a statistically significant relationships between the TBW and the “Cognitive-Perceptual” domain of the SPQ. (**B**) The figure shows a statistically significant relationships between the simultaneity rate and the “Cognitive-Perceptual” domain of the SPQ.

The analysis of the Pearson correlation coefficient between measures derived from the SJ task and the SPQ scores (Fig. 4) showed: (i) a significant positive correlation between TBW and the "Cognitive-Perceptual" subscore of the SPQ (r = 0.281, p = 0.033); (ii) a significant positive correlation between the simultaneity rate and the "Cognitive-Perceptual" subscore of the SPQ (r = 0.261, p = 0.045). All correlational analyses were subject to a Benjamini–Hochberg correction to check for multiple comparisons.

As predicted based on previous studies, the pattern of correlations is consistent with a lower multisensory temporal acuity, as indexed by the raw simultaneity rates and TBW obtained from the psychometric curve, in the presence of more schizotypal traits.

## Discussion

The current study represents a first empirical report on the possibility of adequately administering a SJ paradigm through a web-based platform. Our results confirmed that the response pattern of perceived simultaneity of audiovisual stimuli varies according to the time interval between the two stimuli, with simultaneity reports more common when the SOAs between the auditory and visual stimulus was brief, and a clear decline in perceived simultaneity at longer SOAs. This pattern of results is in line with classic lab-based studies available in the literature^20,27,52,65^, providing a proof-of-concept that the results obtained from the present web-based SJ task closely mimic those obtained in controlled laboratory contexts. Such a finding opens up a realm of potential new studies that target under-represented participant populations (e.g., not just university students) and big data approaches with large sample sizes.

While the influence of the critical role of the temporal relationship between incoming audiovisual stimuli on multisensory integration has been extensively studied (for a review, see^79^), less is known about how the leading sense influences multisensory temporal acuity. To further investigate this question, we separately analyzed the left side (AL) and the right side (VL) of the psychometric curve to assess whether the individual thresholds differed as a function of the leading sense, and whether this also interacts with the spatial congruency factor. We did not find significant evidence that the spatial congruency factor modulated simultaneity rates and/or thresholds in the present online paradigm, although the visual comparison of the simultaneity rate curves in AL trials seems to suggest a rightward shift of the psychometric curve when the presentation of the audiovisual stimuli was spatially congruent compared to the condition of spatial incongruency, coherently with the previous literature. The lack of a significant effect in the present online task could be due to the use of headphones/earphones. This strategy was adopted in order to be sure that auditory presentation was entirely lateralized, something that could hardly be obtained with laptop speakers. However, given that the spatial location of auditory and visual stimuli is vital for eliciting a spatial congruency effect^20,27^, this choice could have been suboptimal.

The direct comparison between AL and VL trials, regardless of spatial congruency, revealed significant differences in both thresholds and simultaneity rates, reflecting increased temporal acuity when audition was the leading sense. The replication of this common pattern in a web-based platform is noteworthy, considering that the neurocomputational processes underlying cross-modal integration in AL and VL trials might be substantially different. Accordingly, recent evidence has shown that the distribution obtained from the simultaneity rate leads to a narrower size of the TBW in AL trials, and a broader size for VL trials, suggesting that such integrative processes are not symmetrical, and that temporal binding of audiovisual information is regulated by two distinct mechanisms of multisensory interactions that depend on the order of presentation of these stimuli^21,80^. In support of these asymmetries highlighted at the behavioral level there are also studies showing that the leading sense leads to markedly different neural processes, identifiable in different EEG/ERP topographical patterns for AL and VL trials, in support of a dual-route model of audiovisual integration that would depend on the leading sense^45^.

As suggested by Cecere and colleagues^44^, this neurocognitive asymmetry between AL and VL in the temporal process of multisensory integration could be explained by the processes of a cross-modal phase reset of brain oscillations in certain frequency bands, in which the sampling mechanisms of one sensory modality interacts with the other modality^81–83^. Critically, for multisensory processes related to audiovisual stimuli the nature of cross‐modal phase reset depends on the leading sense^84^. Typically, in the AL condition, the phase reset process signals the pre-imminence of an input to the visual system, by means of an attentional low-level mechanism, capable of explaining the efficiency in multisensory integration processes. On the other hand, in the VL condition the phase reset process seems to be driven by higher‐level prediction mechanisms, which results in a lower accuracy in the judgments of asynchrony perception^84^. Hence, the asymmetry between the two leading conditions (AL and VL) in the temporal integration processes revealed by our results supports prior evidence suggesting that the processes underlying the two conditions are at least somewhat independent, highlighting better multisensory temporal acuity for the condition AL, compared to the VL. This result assumes considerable importance in the evaluation of multisensory temporal processes in clinical populations in which these processes are anomalous, favoring the specific identification of sensory/perceptual and cognitive aspects involved in these disorders.

The results obtained here suggest the feasibility of performing a web-based assessment of multisensory temporal integration with a SJ task that has typically been confined to controlled laboratory settings. Considering that the need to expand the average sample size employed in behavioral studies is an essential factor for increasing the validity and reliability of evidence in the psychological science field, the present study brings an example of how use of online psychophysical testing might lead to significant benefits in this sense. While online experimentation has revealed enormous potential for studying large samples of participants in controlled and optimized research settings^72,85^, psychophysical studies online deserve special attention due to their specific nature. Due to the lack of control for the context surrounding an online environment, running psychophysical studies online is particularly challenging given that parameters such as the viewing distance and stimulus size, which are often critical factors in psychophysics experimentation, are not fully controlled. In this regard, new methods are being developed in order to obtain a controlled and reliable experimental context. In a recent study Li and colleagues^86^, for example, introduced a new method capable of estimating the participant's viewing distance by detecting the eccentricity of their blind spot location. This methodological approach is able to automatically adjust and control the geometric configuration of the stimuli based on the estimate of the individual's viewing distance of each participant in the experiment, overcoming the limits deriving from psychophysical experiments conducted online.

Another main purpose of the present study was to address the analysis of the possible link between a higher presence of schizotypal traits investigated with the SPQ, and a lower temporal multisensory acuity indexed by the web-based SJ task. The presence in the non-clinical population of traits belonging to SCZ stimulates the investigation of potential differences in multisensory temporal capacities—typically compromised in patients with SCZ—also in the general population with no known history of this condition. This possibility is suggested by recent evidence that has revealed a strong relationship between impairments in the temporal processes of multisensory integration and the presence of schizotypal traits in the general population^50,63^. In particular, Dalal and colleagues^64^ recently highlighted that individuals with lower multisensory acuity have a higher level of schizotypal traits, especially in the cognitive-perceptual domain. Specifically, we examined the relationship between the different domains investigated by the SPQ and the different measures obtained in the audiovisual SJ task. Our findings, in agreement with previous laboratory-based studies^63,64^, showed that lower multisensory acuity was generally associated with a higher presence of schizotypal traits. When we examined the relationship between the different schizotypal domains and multisensory integration, we found that larger individual TBW and higher simultaneity rates (i.e. decreased temporal acuity) positively correlated with the score obtained in the "Cognitive-Perceptual" subscale of the SPQ. To summarize, our results are in line with the previous literature, supporting the evidence that a lower multisensory temporal acuity is correlated with the presence of more schizotypal traits^50,53,64,87^, which in turn agree with evidence obtained in individuals with a clinical diagnosis of SCZ (^52,56,88^; for a review, see^53^). Our study did not reveal a relationship between schizotypal traits and the existence of asymmetries in temporal multisensory processes associated with the leading sense. However, our preliminary data obtained from the SJ task suggest some specific patterns deriving from the leading sense condition. Thus, it would be interesting to further explore this aspect in SCZ and as a function of the schizotypal traits, and in other clinical populations with known anomalies in multisensory integration such as Autism Spectrum Disorders^41,53^.

Interestingly, the fact that a lower multisensory temporal acuity was selectively associated with an increase in the schizotypal behaviors in the cognitive-perceptual domain connect to some etiological theories of schizophrenia that postulate multisensory integration anomalies as critical factors at the basis of the pathogenesis of the disorder. The "*Panmodal processing imprecision hypothesis of schizophrenia*"^89,90^ suggests that individuals with SCZ possess impairments in the cognitive-perceptual system, which translates into a less accurate processing of sensory information compared to neurotypical individuals. At the neural level, the “*Disconnection hypothesis*"^91^ argues that an aberrant multisensory integration process in this clinical population is produced by an abnormal synaptic connection, and by a lower neural connectivity. Hence, a lower (or atypical) connectivity between the auditory and visual system, could explain the impairment present in the multisensory integration processes since reduced connectivity could lead to slower or less efficient multisensory integration. Such multisensory integration deficits would then lead to a reduced temporal acuity and, in turn, to an inaccurate temporal processing of incoming sensory stimuli, which might play a role in sensory overload and contribute to ambiguous and imprecise perceptual experiences^49,92^; both these characteristics are typically manifested by schizophrenic individuals^53,56^. The link between lower multisensory acuity and higher schizotypal traits in the cognitive-perceptual domain suggest a close relationship between the etiology of SCZ and the ability to integrate inputs from different sensory modalities. Interestingly, the relationship between low audiovisual temporal acuity and high schizotypic traits in the cognitive-perceptual domain have recently been associated with positive symptoms of SCZ^50,93^. In a recent study^64^ the authors investigated this relationship, noting that higher scores in two of the SPQ subscales most reflecting positive symptoms of SCZ (‘Perceptual Experiences’ and ‘Odd Beliefs or Magical Thinking’) are strictly associated with a lower audiovisual temporal acuity in the healthy population, highlighting that the association between temporal acuity and schizotypal traits is not restricted only to unusual sensory experiences.

More broadly, our study supports the findings suggesting that the multisensory temporal integration deficit may be a core symptom and a core etiological factor of the disorder, to which more attention should be paid in the future.

Interestingly, recent evidence has suggested that individuals with higher schizotypal traits tend to have temporal integration deficits even in sensory modalities different from the audiovisual one, bringing new knowledge about the relationship between a general deficit of multisensory temporal acuity and schizotypical symptoms. For example, a wider TBWs in integrating information in the visual-tactile domain has been identified in individuals with high schizotypic traits^94^, and it has been shown that subjects with high schizotypic traits demonstrate stronger susceptibility to the rubber hand illusion^95^. Hence, these findings suggest that in individuals with high schizotypal symptoms the deficits in temporal integration processes are not specific to the audiovisual modality, but may reflect a general deficit in multisensory integration processes.

Thus, multisensory integration might be a useful marker for at-risk individuals that could be identified prior to the typical onset age of schizophrenia. Markers for schizophrenia may be of particular relevance given theories that emphasize the role of epigenetics and environmental stressors in influencing the likelihood that schizotypal traits (and the relevant genes) develop into full schizophrenia (for example^75,76^).

One aspect which would deserve additional investigation is, for example, whether temporal processing is anomalous only in the context of cross-modal integration or whether it could be seen also in unisensory domains, such as the differences in thresholds for segregating two visual flashes as separate percepts^96–99^, an aspect which is still controversial in the literature^52,53,100^. In future work, it would also be valuable to see whether such impairments are amenable to training in order to intervene in clinical populations. There is increasing evidence that multisensory TBWs are at least somewhat plastic and can be narrowed through perceptual learning^14,24,44,101–104^. Even training that is restricted to the visual modality has shown benefits in multisensory temporal processes^14^ i.e. a narrowing of the multisensory TBW. A recent study also demonstrated effects of TBW training at the neural level: individuals who showed a narrower TBW after a perceptual learning training with audiovisual stimuli showed greater activity of the parietal and occipital beta frequencies^105^.

In conclusion, the current findings provide further evidence for the possibility of performing accurate web-based psychophysical assessment using audio-visual multisensory paradigms that were previously limited to laboratory settings. The results obtained also provide additional evidence supporting the leading sense asymmetries in multisensory integration processes. Finally, our pattern of results provides additional evidence in support of previous studies that have highlighted temporal multisensory integration deficits as a hallmark of SCZ and its broader phenotype. Considering that this work represents a first attempt in this direction, further work may move beyond this proof-of-concept finding and extend the investigation to other commonly used multisensory psychophysical tasks, in the hope of establishing the web-based option as a valid alternative to address complex aspects of human multisensory perception outside an experimental psychology laboratory and for a more representative demographic of participants.
